# Experimental Models to Study COVID-19 Effect in Stem Cells

**DOI:** 10.3390/cells10010091

**Published:** 2021-01-07

**Authors:** Rishi Man Chugh, Payel Bhanja, Andrew Norris, Subhrajit Saha

**Affiliations:** 1 Department of Radiation Oncology, University of Kansas Medical Center, Kansas City, KS 66160, USA; rchugh@kumc.edu (R.M.C.); pbhanja@kumc.edu (P.B.); 2 BCN Bio Sciences, Pasadena, CA 91107, USA; andrew@bcnbio.com; 3 David Geffen School of Medicine at University of California at Los Angeles, Los Angeles, CA 90095, USA; 4 Department of Cancer Biology, University of Kansas Medical Center, Kansas City, KS 66160, USA

**Keywords:** SARS-CoV-2, COVID-19, stem cells, organoid system

## Abstract

The new strain of coronavirus (severe acute respiratory syndrome coronavirus type 2 (SARS-CoV-2)) emerged in 2019 and hence is often referred to as coronavirus disease 2019 (COVID-19). This disease causes hypoxic respiratory failure and acute respiratory distress syndrome (ARDS), and is considered as the cause of a global pandemic. Very limited reports in addition to ex vivo model systems are available to understand the mechanism of action of this virus, which can be used for testing of any drug efficacy against virus infectivity. COVID-19 induces tissue stem cell loss, resulting inhibition of epithelial repair followed by inflammatory fibrotic consequences. Development of clinically relevant models is important to examine the impact of the COVID-19 virus in tissue stem cells among different organs. In this review, we discuss ex vivo experimental models available to study the effect of COVID-19 on tissue stem cells.

## 1. Introduction

Coronaviruses are a large group of viruses that can cause serious complications in animals and humans. There are seven classes of coronaviruses that infect people, however, three of these can cause serious, or lethal outcomes in humans. These include severe acute respiratory syndrome or SARS coronavirus (SARS-CoV); Middle East respiratory syndrome (MERS) (MERS-CoV); and, most recently, the new coronavirus severe acute respiratory syndrome coronavirus type 2 (SARS-CoV-2), which has resulted in a pandemic that has infected more than 87 million people and approaching 1.9 million deaths worldwide as of 5 January 2021 (https://www.worldometers.info/coronavirus/), a statistic that is growing daily. Coronaviruses are well known as the common cause of upper respiratory symptoms such as a dry cough, sinusitis, loss of taste and smell, and labored breathing; however, for SARS-CoV-2, a variety of other new and unusual symptoms have also been recognized in both humans [1] and in animal models. This new virus strain emerged in 2019 and, hence, as mentioned, is referred to as COVID-19.

COVID-19 significantly depletes tissue resident stem cell population [2], resulting in impaired tissue regeneration and repair. Moreover, loss of stem progenitor cells triggers the inflammatory and later fibrotic consequences [3,4,5,6]. Therefore, mitigation of tissue stem cell loss should be an effective therapeutic strategy against COVID-19 pathogenesis. In this review, we discuss ex vivo experimental models available to study the effect of COVID-19 on tissue stem cells.

## 2. Experimental Model System for Understanding COVID-19 Pathogenesis

**Figure 1:**
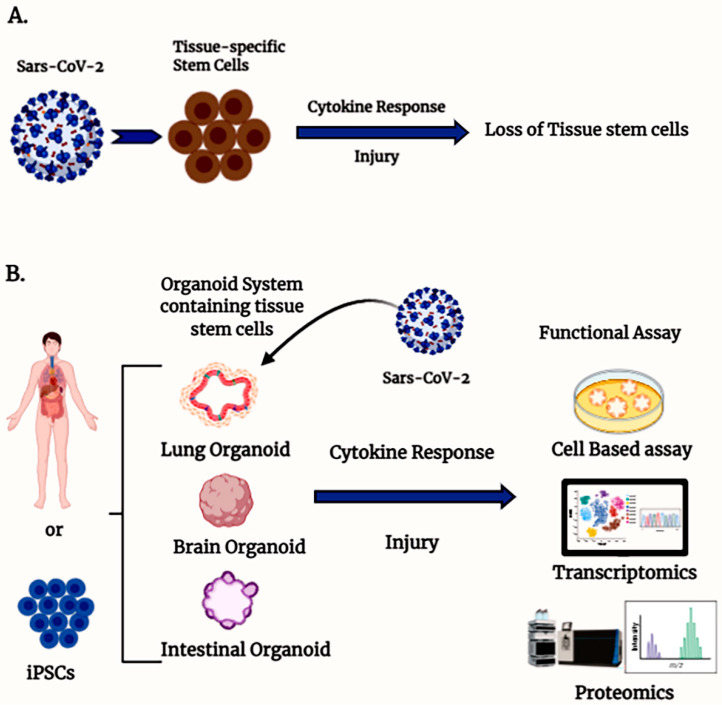
Organoids containing tissue stem cells are an ex vivo model to study severe acute respiratory syndrome coronavirus type 2 (SARS-CoV-2) infection. (**A**) Schematic diagram illustrating the involvement of SARS-CoV-2-mediated stem cell loss. (**B**) Different organ-specific stem cell-derived organoid models to study SARS-CoV-2 infection.

The bulk of our knowledge about the pathogenesis of COVID-19 in humans is based on available clinical trial data and case studies since the outbreak, as well as some preclinical and cell-based testing. The preclinical models available include non-human primates [7] and murine models that express human ACE2 in genetically modified mice such as the K18-hACE2 mice [8], among other tools [9,10], as well as genetically modified virus to recognize murine ACEII [11]. However, there are very limited reports available on a suitable ex vivo model system that can be used to understand the mechanism of action of the virus and can also be used for testing of any drug efficacy against virus infectivity. Tissue-specific stem cell-derived organoid systems could be a better model to understand the effect of COVID-19 on stem cells in the human body (Figure 1).

## 3. Tissue-Specific Stem Cells and COVID Pathogenesis

There are several areas to study that are devoted to the antiviral approach by inhibiting replication (Remdesivir,), building immunity (vaccines), and their impact on the remediation of illness caused by the virus such as acute respiratory distress syndrome (ARDS). Antiviral therapy with Remdesivir has shown promise for reducing recovery time but is not be sufficient to inhibit lethal consequences from infection [12]. Antibody therapies also look highly encouraging if used early in the infection cycle [13], as do vaccines to reduce the spread and severity of the pandemic. Nevertheless, despite these advances, there is legitimate concern that COVID-19 may be present at some level in the population, albeit at a reduced rate, for a long period of time, and for those who contract the virus, long-term or permanent tissue damage is a real possibility in a percentage of cases worldwide.

Previous reports have indicated that in advanced COVID-19 cases, patients possess structural damage in lung epithelium [14,15] and COVID-19 significantly depletes the resident stem cell population [16]. The major pathological outcome due to COVID-19 is the damage to epithelial cells. It is important to note that this is not unique to COVID-19 as it has long been reported that SARS-CoV and H1N1 both propagate within type II cells where a large number of viral particles are released, and the cells undergo apoptosis [17]. These type II cells are presumed to function as progenitor cells that repair the injured alveolar epithelium [18,19,20]. Moreover, damaged epithelial cells also become a major source of inflammatory cytokines that not only can contribute to further damage to the tissue but have systemic and lethal effects as well [21]. Restitution and activation of pulmonary epithelial progenitor cells is critical to inhibit acute inflammation and suppression of pneumonitis/fibrosis (often referred to as ground glass in radiographic images). Loss of these progenitor cells by pathogenic or genotoxic stress impairs the regenerative process, resulting in a reduction in number of healthy epithelial cells, which eventually creates empty space for proliferation and repopulation of newly recruited inflammatory cells [22,23,24]. Moreover, damaged lung epithelial cells release inflammatory paracrine signals to promote recruitment of inflammatory cells. This is also well characterized in early studies with chemical injury as a model to demonstrate that loss of pulmonary stem progenitor cells triggers the inflammatory and later fibrotic consequences [3,4,5,6]. Considering the extensive epithelial damage from COVID-19 virus infiltrate and the impact on lung stem/progenitor populations, further research is warranted and needed to gain a more complete understanding of the pathophysiology of COVID-19 infection. Mitigation of resident lung stem cells may be a key approach to minimize lung damage along with reduction in inflammation and fibrosis. It should also be considered that lungs from recovered COVID-19-infected patients may not regain full structural and functional integrity in severe cases since the repair or rebuilding capacity primarily depends on existing stem/progenitor populations. Early reports indicate lung epithelial stem cells may express SARS-CoV-2 entry factors higher than previously thought [25,26]. Lung contains functionally distinct candidate stem/progenitor cells such as basal cells [27], club cells [28,29], bronchoalveolar stem cells (BASCs) [30], and type II pneumocytes [31] involved in repair and regeneration of injured lungs. In addition to type II pneumocytes, several studies have revealed that a subset of murine and human Oct4+ pulmonary stem cells expressing ACE2 are the prime target of SARS-CoV infection [32,33], which leads to damage and loss of these cells [33].

**Figure 2:**
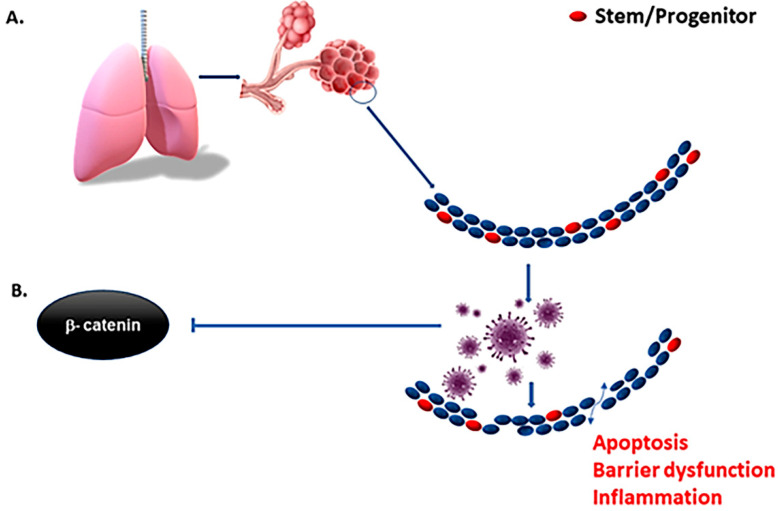
Early inhibition of interferons by SARS-CoV-2 and other viruses serve to suppress the innate immune response, resulting in rapid increases in cellular infection and spread before the adaptive immune response can develop. The depletion of resident stem cells by more virulent forms of viruses impedes the regenerative capacity of the tissue, and in turn increases the inflammatory context (**A**). Virulent forms of influenza suppress β-catenin nuclear localization (**B**) and downstream expression of interferons. Means of mitigating suppression of interferon and the innate immune response in the early phase of infection may reduce viral spread and preserve resident stem cells in the tissue of interest.

As mentioned before, virulent forms of influenza viruses can infect various cell populations in the murine lung, but also display a strong tropism to an epithelial progenitor population defined by the signature EpCam^high^CD24^low^integrin (α6β4)^high^CD200^+^expression. Three-dimensional organoid cultures derived from these epithelial stem/progenitor cells (EpiSPC), and in vivo infection models including transgenic mice, have shown that their enlargement, barrier regeneration, and outcome after virus-induced injury are highly dependent on Fgfr2b signaling. Importantly, virus-infected epithelial progenitor populations exhibited severely impaired renewal capacity due to virus-induced blockade of β-catenin-dependent Fgfr2b signaling, as evidenced by a loss of alveolar tissue repair capacity after intrapulmonary EpiSPC transplantation in vivo [34]. *Wnt* signaling is essential for lung epithelial stem cells repair and regeneration. The *Wnt* signaling pathway was downregulated in both in vivo-infected alveolar epithelial cells and in vitro-infected human lung epithelial A549 cells [35]. These results suggest that the influenza viruses may affect the host lung repair by regulating Wnt/β-catenin signaling. β- and γ-catenin regulate the innate cellular immune response to viruses by activating virus-dependent induction of the IFNB1 and downstream genes. Virulent viruses can suppress β-catenin-dependent transcription by misusing the RIG-I/NF-κB signaling cascade that is induced in the course of infection by viral RNA [36], and we hypothesize that COVID-19 is similar to other viruses in this regard [37]. Therefore, activation of Wnt/β-catenin signaling could be a major therapeutic intervention in the context of viral infection [38] if implemented early in the infectious lifecycle (Figure 2) where the immediate check on viral spread can happen before the adaptive immune response has time to develop several days after infection. More specifically, type I interferons are a critical part of our innate immune defense as they induce an array of proteins that interfere with virus replication in order to restrict and limit viral spread from cell to cell [39] in that early window before the adaptive immune response can even take effect. Viral suppression of this system may lead to unchecked and rapid spread, reaching very high viral loads in the lung and tissues. This, in turn would improve the chances of aerosolization and communication along with extensive tissue damage as the adaptive immune system takes over. While interferons have been used to treat COVID-19 with little success [40], biologically, its expression is timed as an immediate and early response rather than very late advanced disease where clinical trials have focused.

In COVID-positive patients, symptoms are also noted in multiple other organs, most notably the gastrointestinal tract and the kidney. Organoid-based studies demonstrated that SARS-CoV-2 could damage stem cells in these organs. However, the effects of SARS-CoV-2 in different type of stem cells such as intestinal quiescent stem cell populations vs. Lgr5+ active stem cell population is not known. Similarly, effect of SARS-CoV-2 on pancreatic and liver stem cells are predicted but further details are yet to be revealed.

## 4. Human Organoid Systems

The development of clinically relevant models is a critical step to examine the effect of the COVID-19 virus in specific organs. Ex vivo organoid systems have been used extensively to study tissue homeostasis and repair. Moreover, studies related to stem cell homeostasis and/or regeneration are primarily performed in ex vivo organoid systems as stem cells are the building block for organoid survival [41].

The organoid cultures are genetically stable and grow indefinitely [42], in contrast to primary cells or tissue explant models that only offer short-term culture capabilities. These multicellular structures recapitulate many properties of the individual organs, including the heterogeneity of the cellular composition, appropriate physiology, and region-specific features. Additionally, these human tissue-derived cultures allow individual genetic variability, disease status, and other demographic factors including age, gender, and ethnicity. Organoids have also been used to study pathogenesis of micro-organisms [43,44] including viruses [45,46].

Organoid cultures can be derived from either human embryonic stem cells or induced human pluripotent stem cells, or adult stem cells derived from human tissue. RNA-seq analysis demonstrated that organoids exposed to SARS-CoV-2 demonstrated chemokine response such as what is observed in patients. Here, we discuss the relevance of pulmonary, intestinal, neuronal, and kidney organoid system to perform COVID-19 research.

## 5. Pulmonary Organoid in COVID-19 Research

**Figure 3:**
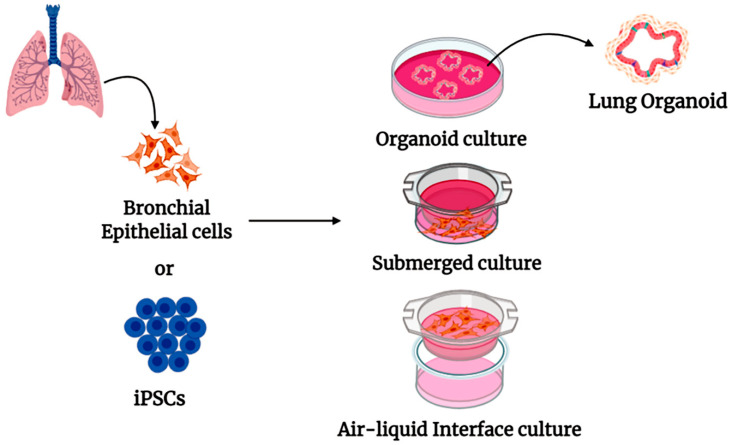
Schematic illustration of isolated lung epithelial cells or induced pluripotent stem cells (iPSCs) for studying SARS CoV-2 virus pathogenesis, using organoid culture, submerged culture, and air–liquid interface (ALI) culture of epithelial cells.

Lung 3D organoids derived from both healthy and diseased lung cells such as bronchial epithelial cells (HBEC), induced pluripotent stem cells (iPSCs), or embryonic stem cells (ESCs) [47,48,49] have been used to determine lung biology, diseases, and treatment response. This model has also been used to study virus pathogenesis and pulmonary fibrotic lung disease [50]. Although attempts have been made for long-term expansion of pseudostratified airway organoids [51], the presence of the inner lumen (facing inwards) makes stimulation and collection of the sample more challenging. The air–liquid interface (ALI) model has been considered as an alternative to this system (Figure 3). In ALI models, iPSC cells or lung epithelial cells are enlarged to merge into an inaccessible filter [52], and therefore the media can be removed from the apical side of the filter. This system allows cells in contact with air and enables the cells to divide into a mature phenotype including pseudo-stratified epithelium, consisting of functional basal, ciliated, and secretory cells. Several reports confirm that structure, function, and genetic profiles of ALI lung model are very similar to nasal or bronchoscopically obtained tracheal and bronchial brushings from human airways [53,54]. The ALI model consists of both apical (upper) and basal (lower) chambers suitable for any treatment and sample collection. The ALI model is also suitable for determining epithelium integrity, mucociliary clearance, and cilia beat frequency [55,56]. SARS-CoV-2-mediated epithelial cell proinflammatory response as well as therapeutic response of Remdesivir has been studied in ALI culture. Therefore, ALI cultures can be considered as the most appropriate ex vivo model to study COVID-19 pathobiology and robust screening of potential anti- SARS-CoV-2 candidate agents.

However, the absence of stroma and immune cells are one of the major limitations of the organoid system in SARS-CoV-2 research. SARS-CoV-2 infections result in a complex respiratory disease including epithelial damage and a dramatic inflammatory response. The lung-on-a-chip model provides a small dynamic living and biological environment, consisting of a 3D cell culture system divided by a dense membrane, consisting of channels that allow continuous perfusion to mimic circulation in the body carrying major immune cell types, as well as cleansing chambers that mimic breathing in human lungs [57]. Various microsensors within the microchip enable real-time data collection, such as barrier function, surfactant production, protein production, fluid pressure, and cell migration [58]. Organ-on-a-chip models have thus been able to replicate in vivo-like environments and can allow for the comparison of biological responses under normal and disease conditions [59]. A list of observations using the lung organoids to study SARS-CoV-2 are presented in Table 1.

## 6. Intestinal Organoid Models

Patients with COVID-19 experience gastrointestinal symptoms, such as diarrhea, nausea, vomiting, and loss of appetite [60,61]. ACE2 and TMPRSS2 were co-expressed in esophageal, upper epithelial, and gland cells and in absorptive enterocytes from the ileum and colon [61], therefore playing key role in viral entry possibly explaining why diarrhea is one of the early symptoms of COVID-19 infection.

Intestinal organoid models derived from both mouse and human tissue have been used extensively to study viral pathogenesis. Several human intestinal viruses, including rotavirus, norovirus, enterovirus, adenovirus, and coronavirus, have now been demonstrated to infect human intestinal organoid cultures [62,63,64,65]. The human organoid system can be developed from both adult tissue stem cells and induced pluripotent stem cells. High levels of ACE2 expression and viral RNA have been detected in anal swabs, stool, and sewers, suggesting susceptibility of intestinal epithelium for significant COVID-19 infection. Studies [65,66] using human multipotent adult tissue stem cell-derived intestinal organoids reported that the most common cell type of the intestinal epithelium, the enterocyte, is readily infected, suggesting that the intestinal epithelial cells are one of the highest infection locations for SARS-CoV-2 virus. Upregulation of viral response genes were observed in infected enterocytes, possibly through cytoplasmic sensing of the viral RNA genome (Table 1) [67]. In addition, the presence of membrane-bound serine proteases TMPRSS2 and TMPRSS4 in intestinal epithelial cell cleaves the SARS-CoV-2 spike protein to facilitate viral entry [68]. Intestinal organoid survival, much like intestinal crypt architecture, is also a stem cell-driven process and primarily depends on Wnt/beta catenin signaling. Intestinal tissue consists of quiescent stem cells and active stem cells. ACE2 expression level in these two types of stem cells and their susceptibility to COVID-19 infection are important to examine in terms of the development of a potential therapeutic target. Involvement of beta catenin signaling in inhibition of viral propagation makes it more important to investigate the role of Wnt/beta catenin signaling in intestinal stem cell response against COVID-19 infection.

## 7. Neuronal Organoid Models

Emerging case reports have shown that patients infected with SARS-CoV-2 suffered severe neurological symptoms including sudden and complete loss of the olfactory function, stroke, seizure, encephalopathy, encephalitis, Guillain–Barré syndrome, and Miller Fisher syndrome [69,70,71,72,73], along with pathognomonic symptoms of anosmia (loss of smell) and ageusia (loss of taste). All of these indicate that SARS-CoV-2 could infect the central nervous system (CNS) and is therefore neurotropic [74,75]. Postmortem brain MRI analysis has identified the presence of hemorrhagic and encephalopathy syndromes, suggesting that SARS-CoV-2 infection could cause neuronal stress and inflammation [76]. SARS-CoV-2 has been reported to infect nerve cells, for example, neurons in the medulla oblongata, which is part of the brain stem that serves as the control center for the heart and the lungs, with the damage potentially contributing to “acute respiratory failure of patients with COVID-19” [77]. These studies have shown that SARS-CoV-2 can infect neurons and cause neuronal death in an ACE2-dependent manner [77]. In brain cells derived from human pluripotent stem cells, microglia and cortical neurons were not infected, however, dopaminergic neurons were highly susceptible to SARS-CoV-2 infection [78]. In humans, however, viral load in neuronal tissue appeared to be at a low enough level to evade detection, even if there was encephalitis or CSF inflammation [79,80,81,82]. Thus, further work needs to be done to establish neural cell targets in humans, but the organoid studies may be an informative model to enhance our understanding. Human neuron progenitor-derived spheroids or organoid cultures have been used as a model for several years now to study neuro-degenerative diseases and for screening potential therapeutic effects. Organoids derived from iPSCs exhibiting a wide diversity of cell types could serve as a suitable model system to test the neurotoxic effects of SARS-CoV-2 [68,83,84,85,86]. Organoid-based data have revealed that SARS-CoV-2 exposure is associated with altered distribution of Tau from axons to soma, hyperphosphorylation, and apparent neuronal death. A human brain organoid study showed clear evidence of infection with accompanying metabolic changes in the infected and neighboring neurons, which can be prevented either by blocking ACE2 with antibodies or by administering cerebrospinal fluid from a COVID-19 patient. It has been observed that cells dying within the organoids are sometimes a neighbor to the infected cells, suggesting a possible bystander effect of COVID-19 infection. Compared to other neurotropic viruses, SARS-CoV-2-infected brain organoid demonstrates modulation of pathways related to cell division, organelle fission, and metabolic processes. Therefore, it is very clear that more studies are required to determine the neurotrophic effect of SARS-CoV-2 where organoids will be one of the most robust ex-vivo models due to its relevance to COVID-19 infection in the human nervous system.

The major limitation of these neuronal organoid models is the absence of vascularization as in adult brain. Blood vessels are critical for gas exchange, nutrient supply, and waste removal, and may possibly present physical differences to organoid cultures. In addition, introduction of mesenchymal cells or iPSC-derived endothelial cells will be more pertinent to develop neuro organoids mimicking in vivo cerebral system. The presence of myeloids such as microglia will be also very critical to reproduce the cerebral micro-environment. Therefore, further improvements in organoid model system are also needed to resemble the diversity of cell types and facilitate connectivity between different regions of the brain. An important finding using the neuronal organoids model are summarize in Table 1.

## 8. COVID-19 and Kidney Injury

Kidney disease has been found to be associated with a worse outcome from COVID-19 infections [87], and this is attributed to a variety of conditions such as hypovolemia, acute respiratory distress syndrome, cytokine storm, and direct viral invasion [88]. A detailed study in the United Kingdom [89] looking at data from ICUs between 10 March and 31 July 2020 found that of the 372 patients studied, 216 (58%) had kidney impairment, 22% of which was pre-existing chronic kidney disease and 78% of which developed during their hospitalization from COVID-19. Importantly, it was found that patients with non-detectable kidney damage (21%) died, and those patients with kidney disease developed during their hospitalization (48%) died, indicating the kidney is a prominent target of COVID-19. Although still under investigation, it is important to point out the fact that according to the Human Protein Atlas [90] (http://www.proteinatlas.org), for both entry factors, ACE2 and TMPRSS2, kidney represents one of the highest expression levels of any organ in the body. Because the virus needs these entry factors to infect cells, it is conceivable that viral invasion may be a significant contributor to kidney damage. These same receptors are on cells of the lungs and heart where COVID-19 has been shown to cause tissue damage. In kidney, tubule epithelial cells and podocytes are enriched in ACE2 and TMPRSS2 [91,92]. It has been reported that pro-inflammatory and profibrotic processes in the kidney following COVID-19 infection are primarily due to internalization of ACE2, resulting in imbalance in the renin–angiotensin–aldosterone system, with increased Ang II signaling [93,94]. Electron microscopy examination of autopsy samples from 26 patients demonstrated clusters of viral particles in the tubular epithelium and podocytes, suggesting SARS-CoV-2 exerts tropism in the kidney. In vitro studies using kidney organoids demonstrated that SARS-CoV-2 infection can be minimized by human recombinant soluble ACE2 [95].

From this study we can summarize the different organoid models used in SARS-CoV-2 study (Table 1).

**Table 1:** Summary of organoid models in SARS-CoV-2 study.

Organoid Model	Observation/Findings	References
Human adult tissue stem cell-derived intestinal organoids	SARS-CoV-2 infects human gut enterocytes and replicates to increase viral pool in intestine. Mature enterocytes are susceptible to SARS-CoV-2 infection as they are enriched in angiotensin-converting enzyme 2 (ACE2) viral receptor. Membrane-bound serine proteases, TMPRSS2 and TMPRSS4, expressed in enterocytes and promote virus entry.	[65,66,67]
Lung organoid	Determinations of SARS COVID-2 pathology. Lung stem cell response to SARS-CoV-2. Androgen signaling regulates ACE2 expression in alveolar epithelium. Downregulation of lipid metabolism in lung epithelium with SARS-COVID-2 infection. Screening of SARS-COVID-2 inhibitors. Three entry inhibitors were identified: imatinib, mycophenolic acid, and quinacrine dihydrochloride.	[96,97,98,99,100,101,102]
Neuronal organoid models	Analysis of ACE2 and TMPRSS2 expression in brain organoid. Neurotoxic effect of SARS-CoV-2. SARS-CoV-2 damages the choroid plexus epithelium. Resulting loss of barrier and allowing entry of pathogens, immune cells, and cytokines into cerebrospinal fluid and the brain. Sofosbuvir, an FDA-approved antiviral drug, protects brain organoid from SARS-CoV-2.	[103,104,105,106]
Kidney organoid	SARS-CoV-2-associated acute kidney injury. Combination therapy using Remdesivir with recombinant soluble ACE2 (high/low dose) reduces virus entry and replication. Human recombinant soluble ACE2 inhibits SARS-CoV-2 infection and mitigates propagation.	[107,108,109]

## 9. Conclusions

The inflammatory response among other long-term consequences are a major topic of research on COVID-19. However, the involvement of tissue stem cells in COVID-19 pathogenesis is very important to understand, and further research is needed to determine their role. This review highlights the current models available for SARS-CoV-2 effects as it relates to stem cells. While SARS-CoV-2 infection can result in a complex multi-organ syndrome, stem cell-based models from multiple impacted organs are essential and well suited for the purpose, as ex vivo organoid models are widely accepted for stem cell research in general. SARS-CoV-2 infection, however, involves multiple cell types and their interactions with stem cells. Therefore, more complex multicellular organoids or organ-on-a-chip technologies may be more advantageous in examining SARS-CoV-2 infection and stem cell response.

## Data Availability

Not applicable.
